# Bioprecipitation of Calcium Carbonate Crystals by Bacteria Isolated from Saline Environments Grown in Culture Media Amended with Seawater and Real Brine

**DOI:** 10.1155/2015/816102

**Published:** 2015-07-26

**Authors:** G. A. Silva-Castro, I. Uad, A. Gonzalez-Martinez, A. Rivadeneyra, J. Gonzalez-Lopez, M. A. Rivadeneyra

**Affiliations:** ^1^Department of Microbiology, Faculty of Farmacy, University of Granada, University Campus of Cartuja, 18071 Granada, Spain; ^2^Department Civil Engineering, School of Civil Engineering, University of Granada, Campus of Fuentenueva, 18071 Granada, Spain; ^3^Department of Electronic and Computer Technology, School of Computer Sciences and Telecomunications, University of Granada, University Campus of Almanjayar, 18071 Granada, Spain

## Abstract

The precipitation of calcium carbonate and calcium sulphate by isolated bacteria from seawater and real brine obtained in a desalination plant growth in culture media containing seawater and brine as mineral sources has been studied. However, only bioprecipitation was detected when the bacteria were grown in media with added organic matter. Biomineralization process started rapidly, crystal formation taking place in the beginning a few days after inoculation of media; roughly 90% of total cultivated bacteria showed. Six major colonies with carbonate precipitation capacity dominated bacterial community structure cultivated in heterotrophic platable bacteria medium. Taxonomic identification of these six strains through partial 16S rRNA gene sequences showed their affiliation with Gram-positive *Bacillus* and *Virgibacillus* genera. These strains were able to form calcium carbonate minerals, which precipitated as calcite and aragonite crystals and showed bacterial fingerprints or bacteria calcification. Also, carbonic anhydrase activity was observed in three of these isolated bacteria. The results of this research suggest that microbiota isolated from sea water and brine is capable of precipitation of carbonate biominerals, which can occur *in situ* with mediation of organic matter concentrations. Moreover, calcium carbonate precipitation ability of this microbiota could be of importance in bioremediation of CO_2_ and calcium in certain environments.

## 1. Introduction

Carbon dioxide (CO_2_) can be released to the environment from fixed sources (such as power generation facilities of industries) and from mobile sources (such as cars and trucks). The Intergovernmental Panel on Climate Change Special Report on Emission Scenarios [1] estimates CO_2_ emissions to be as high as 29–44 Gt per year in 2020 and 23–84 Gt per year by 2050. It is estimated that 60% of fixed emissions occur at specific locations and in a large-scale scenario. Most can be remediated by CO_2_ sequestration and storage, which also incurs in economic cost [2].

Different technologies can be applied for CO_2_ capture and store [3]. In this context, one form for CO_2_ capture is the geological carbon sequestration (GCS), which stores CO_2_ for indefinite periods of time at geological sites such as deep saline aquifers or depleted oil or gas wells. This is particularly attractive due to the possibility of usage of fossil fuels and, at the same time, consideration is paid to global warming phenomenon. However, there may be some doubts about the application of GCS. One of the most important concerns is the CO_2_ leakage through wells, faults, or fractures in the low-permeability cap rock [4]. Risk of leakage is a more pressing concern given that scCO_2_ is less dense and less viscous than natural pore fluid, which offers the possibility of CO_2_ upwards migration through sequestrating geological formation [5]. However, biological approaches such as the use of engineered biofilms or biomineralisation processes have been proposed to mitigate leakage and enhance CO_2_ storage [6].

It has been also proposed that the bioprecipitation of minerals such as calcium carbonates could be considered a new strategy to remove carbon dioxide or to prevent its emission [7]. In this context, several possible mechanisms of microorganisms-mediated mineral precipitation in natural and engineered environments have been proposed [8–10]. In spite of this, the role that microorganisms have in the biological process and their influence over the crystal characteristics of precipitates is still not understood [11]. Thus, certain microbial species have been found to be associated with biomineral precipitation in many different environments such as saline habitats (seawater, brine), biofilms, and soils [12, 13]. Relationships between microorganisms species and biomineral characteristics have been suggested [11], although the biological precipitation mechanisms as well as the impact of this process in the microbial ecology of precipitating organisms are still unknown.

Several studies have reported the bacterial precipitation of carbonates [9, 10, 14] and have suggested different mechanisms for the microorganism-mediated precipitation of carbonate biominerals [15, 16]. The formation of calcium and magnesium carbonates has been explained by the production of NH_4_^+^ and CO_2_ in presence of calcium and magnesium ionic species. Other authors have proposed that adsorption of calcium, magnesium, and metallic cations to cell surface could trigger bacterial-mediated biomineralization, with bacterial cell serving as nucleus for precipitation; in this sense, characteristics of extracellular polymeric substances impact biomineral precipitation [15, 16]. However, the influence of abiogenic features over bioprecipitation process is still unresolved, and thus optimal microbially mediated precipitation conditions are still not known. Among these abiogenic factors, the ionic composition of the medium and its salt concentrations are thought to be the most important factors in carbonate minerals bioprecipitation, along with fluid chemistry and fluid flow or viscosity [17].

Halophilic bacteria or microorganisms that have adapted to high-range salinity-changing environments can be used for the determination of the effect of the ionic characterization of the medium over the bacterial carbonate mineralization and therefore over CO_2_ sequestration [10]. Furthermore, previous studies have reported mineralogical differences of biominerals formed by precipitation mediated by halophilic bacteria [10, 18] as well as the different roles that these bacteria play in carbonate mineralization at both natural environments and laboratory culture media.

As reported above, and considering the amounts of CO_2_ in the atmosphere, one alternative is to create new equilibrium conditions in calcium and carbon cycles by storing and sequestering CO_2_ in the stable form of calcium carbonate [7]. As a result, research is now being carried out regarding the use of microorganisms to produce calcium carbonate precipitations that are extremely stable in certain environments. In this context, carbonic anhydrase (CA) enzyme catalyses the reversible hydration of CO_2_ in many organisms. Even though the enzyme has been found in many environments, its precise functions and mechanisms of action are not well known [19]. CA has an important role in carbonate precipitation in different marine organisms [20] and algae [21] and in the formation of calcified structures of corals and diatoms [22]. The presence of CA in metabolically diverse species within both* Bacteria *and* Archaea* domains is indicative of the significant role of this enzyme in prokaryotic microorganisms [23], even though little is known about its function. Sánchez-Moral et al. (2003) [24] suggest that CA could also be responsible for the capture of an important fraction of carbon dioxide by heterotrophic bacteria in underground environments.

In this paper, we studied biomineral precipitation by different bacteria isolated from saline environments and its capability for biomineral formation including calcium carbonate in both liquid media and solid media. The morphology, mineralogy, and texture of the precipitates in laboratory cultures, amended or not with brine obtained from a desalination plant as the only calcium source, were also studied. Finally, the role of the carbonic anhydrase (CA) in carbonate precipitation and capture of CO_2_ was determined and discussed.

## 2. Materials and Methods

### 2.1. Bacterial Strains

The experiments were performed with bacterial strains isolated from natural brine produced in a desalination plant located in Ibiza Island (Spain) and from seawater samples obtained from the Mediterranean Sea in the area close to the desalination plant. The isolation, selection, and study of capacity of these bacterial strains for biomineral precipitation were carried out in a specific medium for bacterial carbonate precipitation. The medium M1 composition (wt/vol) was the following: 1% yeast extract, 0.5% protease peptone, 0.1% glucose 0.4% calcium acetate, and 20 g/L Bacto-Agar and a mixture of sea salts to achieve a 3% final concentration (wt/vol). 1 M KOH was used to set the pH to 7.2.

0.1 mL brine or seawater dilutions in sterile saline solution were used for the inoculation of M1 medium plates (5 plates per dilution). Inoculated plates were incubated under aerobic conditions at 25°C temperature. Total heterotrophic aerobic bacteria were counted. Also, all plates were examined through optical microscopy periodically to check the presence of crystals, and the crystal-forming and non-crystal-forming colonies were counted. Isolated representatives of the dominant colony morphologies (3 major colony types from brine samples and 3 major colony types from seawater samples) were selected and purified, by restreaking them.

### 2.2. Identification of Selected Strains

All of the selected strains (with carbonate precipitation capacity) were taxonomically classified by analysing the partial sequence of the gene encoding 16S rRNA. DNA extraction procedures, PCR amplification process, purification techniques, and sequencing methods were done following Rivadeneyra Torres et al. (2013) [13]. Phylogenetic affiliation of isolated strains was done using the hypervariable V1–V3 (650 bp) regions of the sequenced 16S rRNA gene. Bioinformatic processing of isolated strains partial 16S rRNA gene corresponding to sequence similarity searching, phylogenetic analysis, and phylogenetic tree conformation was done in accordance with Rivadeneyra Torres et al. (2013) [13].

### 2.3. Study of Carbonates Formation

In order to study whether brine may be a suitable source of cations for the carbonate, bioprecipitation experiments with the selected bacterial strains were performed in solid and liquid media. In this context, the chemical composition (data reported by Aqualia, Ibiza, Spain) of the brine used in our study was as follows (mg/L): Ca^+2^ 770; Mg^+2^ 2340; Na^+^ 11800; K^+^ 450; Cl^−^ 21100; SO_4_^−2^ 3166; HCO_3_^−2^ 238; pH 6.97.

#### 2.3.1. Carbonate Crystal Formation in Solid Medium

Solid media used for carbonate precipitation were composed of real brine amended with different amounts and sources of organic matter, as indicated in Table 1. The main purpose of the selection of these different culture media for the study of the carbonate precipitation was to evaluate the effects that different concentrations and types of carbon sources have on the crystal formation. For the purpose of solid media conformation, 18 g/L of Bacto-Agar was added, while pH was set to 7.2 by 0.1 M KOH addition. The media was then autoclaved during 20 minutes at 112°C temperature.

The strains were inoculated onto different solid media and incubated under aerobic conditions at 25°C temperature. Optical microcopy was used for periodical examination of crystal presence in the media up to the 30 days after media inoculation. pH-indicator paper was used for the measurement of pH at the end of both growth and mineral formation experiments. After 30 days of incubation, precipitates produced by all strains were collected from solid culture media and studied by X-ray diffraction (XRD). The experiments were carried out in triplicate and were repeated three times. Control experiments consisting of media inoculated with autoclaved cells and of noninoculated media were also conducted.

#### 2.3.2. Liquid Media

Two selected strains (S3 and M3) were inoculated into two Erlenmeyer flasks of 1 L containing 200 mL of SM2 medium (Table 1). SM2 medium was selected for this study because an optimal condition for the formation of carbonate crystals was detected. Culture media were incubated at 25°C under aerobic conditions during 30 days. The dynamics of the following parameters was monitored: pH, Ca, and Mg ions concentrations; total organic carbon (TOC); and inorganic carbon (IC). The pH was measured with a pH Meter Basic 20 (Crison); Ca_2_^+^ and Mg_2_^+^ were measured with a Perkin-Elmer 5100ZL atomic absorption spectrophotometer with flame photometry, graphite camera, and automatic sample analyzer equipment; TOC and IC were measured with a Rosemount Analytical TOC Analyzer. Precipitates after 30 d were collected following the methods described in Rivadeneyra et al. (2004) [18]. Control experiments were conducted using autoclaved cells as inoculum and using noninoculated culture medium.

### 2.4. Mineralogical Study

X-ray diffraction examination of obtained minerals from both solid and liquid culture media was done following Rivadeneyra Torres et al. (2013) [13] using the XPowder software [25, 26].

### 2.5. Morphological Studies

Observations and morphologic studies of the bacterial precipitates obtained were also carried out by scanning electron microscopy (SEM), using an LEO 1430VP and an LEO 1430VP equipped with an EDX system INCA350, or a Zeiss DMS scanning electron microscope. In order to provide better resolution images, the samples were golden and covered with carbon to carry out EDX microanalysis. In order to obtain high resolution images, we used a scanning electron microscope (FESEM) 2-3 kV LEO 1525 and samples covered with carbon.

### 2.6. Carbonic Anhydrase Assay

A carbonic anhydrase (CA) assay was performed, following Ramanan et al. (2009) [27]. The reaction is based on hydration, in the presence of the CA enzyme, of p-nitrophenyl-acetate (p-NPA) to p-nitrophenol and acetate, which produces a yellow coloration. Bacterial strains were inoculated onto TSA agar and incubated at 28°C for 24 hours over a period of five days. The plates were subsequently sprayed with a solution of 10 mM of p-NPA (p-nitrophenyl acetate), and the positive colonies showed yellow colour zones.

### 2.7. Geochemical Study

PHREEQC software version 2.17.01 [28] was used to determine the activity of the dissolved species and saturation degree in the initial solutions assayed. Total phosphorous was measured using the colorimetric analysis in the nitrogen digests. The total carbon and nitrogen in culture media were measured by Elemental Analysis with an organic elemental analyser that had a thermal conductivity detection system (Thermo Scientific Flash 2000). In solid media, values of C, P, and NH_4_^+^ were calculated on the assumption that all of the organic substrates added had been metabolised. The C, P, and N data in the culture solutions correspond to the values of the metabolised organic matter in the cultures of the bacterial strains. All of the calculations were performed with the values presented in Table 2.

## 3. Results and Discussion

The number of heterotrophic bacteria (CFU) per mL brine and seawater samples was in the range of 5.5 × 10^5^ and 2.8 × 10^6^, respectively. The carbonate-forming bacteria percentage in M1 soil medium was 85% in brine samples and 94% in seawater samples. The role of microorganisms in biomineral precipitation has been reported for different mineral formation processes. In this sense, several researches have highlighted the role of microorganisms in mineral precipitation in natural ecosystems [29, 30]. In general, biomineralization does not have to be related to any specific microbial group, even though when these bioprocesses have been reported in numerous ecosystems. In this context, our results showed that both seawater and brine samples presented higher bacterial populations with the ability to precipitate carbonate crystals in culture media containing real brine as a source of calcium and magnesium.

**Figure 1:**
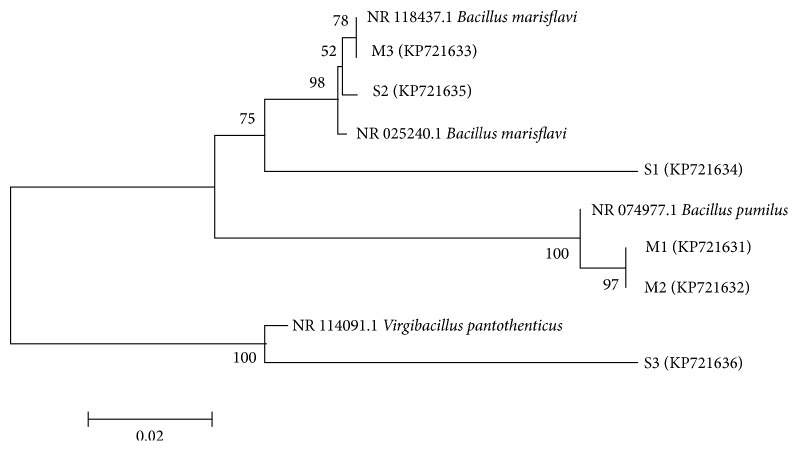
Phylogenetic tree of the six bacterial isolates based on 16S rRNA gene partial sequences.

Six major colonies with the greatest production capacity of crystal formation (three from brine samples and three from seawater samples) were selected for taxonomic identification and the study of crystal formation using brine as a calcium source. The taxonomic identification of selected strains is shown in Table 3. Isolated bacterial populations were closely related to Firmicutes phylum, according to the phylogenetic tree (Figure 1). Specifically, all isolates with the ability to precipitate minerals were classified into two closely related genera* Bacillus* (5 strains) and* Virgibacillus* (1 strain). BLAST search of selected strains sequence showed that strain M1 was related to* Bacillus pumilus *(99% identity), strain M2 to* Bacillus pumilus* (99% similarity), strain M3 to* Bacillus marisflavi *(97% identity), strain S1 to* Bacillus marisflavi* (91% similarity), strain S2 to* Bacillus marisflavi* (95% identity), and strain S3 to* Virgibacillus pantothenticus* (89% similarity). It has been found that microorganisms belonging to the Firmicutes phylum are the dominant phylotypes involved in carbonate precipitation in extreme environments [31], while Bacillales-related bacteria have been reported for their role in organic matter hydrolysis and biodegradation.

The capability of the selected strains to bioprecipitate carbonate in solid media with added brine and its carbonic anhydrase activity is shown in Table 4. The incubation time required for the formation of crystal carbonates by the bacterial strains was affected by the particular strain and culture medium used. Thus, crystal formation by M3 and S3 strain growth in SM1 and SM2 medium took place rapidly, beginning at 2 days after inoculation, while M3 and S3 strain growth in SM5 and SM6 medium formed carbonate crystals after 14 days of inoculation. Obviously, after this period of time, all of the strains formed larger amounts of crystals both in quantity and in size.

Regarding the carbonic anhydrase activity (Table 4), it was observed that three strains (M1, M2, and M3) isolated from seawater samples showed an intense yellow colour when its colonies were sprayed with a solution of 10 mM p-NPA, indicating a positive reaction to the presence of the enzyme. However, the strains isolated from brine samples (S1, S2, and S3) did not reveal any carbonic anhydrase activity.

All of the strains selected from seawater samples showed CA activity while the strains selected from real brine had negative enzymatic activity (Table 4). Liu et al. (2005) [20] reported that CA catalyses the reversible hydration balance from carbon dioxide to bicarbonate in many organisms, which decisively influences biological carbonate precipitation in different types of organisms, both prokaryotic and eukaryotic. In the same sense, Sánchez-Moral et al. (2003) [24] concluded that CA could also be responsible for the sequestration of CO_2_ by heterotrophic bacteria. In addition, scientists have now begun to study the potential use of CA for the storage and capture of CO_2_ through its precipitation in the form of insoluble carbonates [7, 27]. In our study, CA activity was detected in three of the six strains, with the capacity to precipitate calcium carbonate; consequently, it might be suggested that this enzyme could not affect carbonate precipitation. However, more experimental data is needed in order to confirm this preliminary hypothesis, since other factors can probably affect this biomineralisation process. Nevertheless, the efficient ecological or industrial application of carbonate precipitation by bacteria or carbonic anhydrase activity requires greater knowledge of these processes and the action of the enzyme that characterises them. In this context, more experiments are in progress.

**Figure 2:**
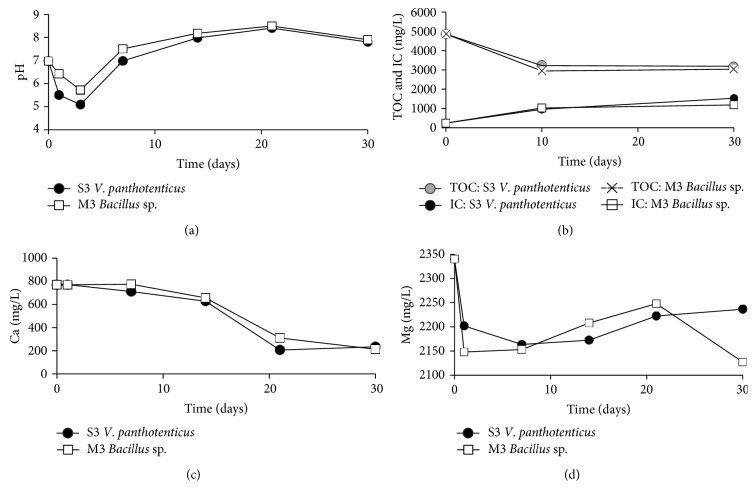
pH (a), total organic carbon and inorganic carbon (b), calcium concentration (c), and magnesium concentration (d) in SM2 liquid medium inoculated with* Virgibacillus pantothenticus* S3 and* Bacillus marisflavi* M3.

Figure 2 shows the values of pH (a), total organic carbon and inorganic carbon (b), calcium concentration (c), and magnesium concentration (d) during the growth of S3 and M3 strains in SM2 liquid medium.* Virgibacillus pantothenticus* S3 and* Bacillus marisflavi* M3 initially decreased the pH of the culture medium at values of 5.0 and 5.8, respectively. However, after 4 days of incubation, the pH values in the liquid media were slowly increased until values became close to 8.0 at the end of the experiments.* Virgibacillus pantothenticus *S3 decreased calcium, magnesium, and organic matter concentrations at values of 69.5%, 4.4%, and 34.5%, respectively, while* Bacillus marisflavi* M3 reduced concentrations of calcium, magnesium, and organic matter 72.3%, 9.1%, and 37.6%, respectively. Obviously, changes in pH, TOC consumption, calcium, and magnesium were not detected in the control experiments.

The metabolic activity of bacteria has an important role in the mineralisation process. The pH of the liquid cultures (Figure 2) as well as solid media increased significantly from a pH value of 7 to a value of 8. The reduction of organic carbon in the cultures of the selected bacteria strains was approximately 40% (*Bacillus* and* Virgibacillus*). In this sense, according to previous research [8], the release of CO_3_^2−^ and NH_4_^+^ ions (with an increasing pH), in the presence of Ca^2+^ or Ca^2+^ and Mg^2+^ ions, produces carbonate precipitation. In our culture media, which contained high concentrations of Ca^+2^ and Mg^+2^ ions and high concentrations of organic matter (in the forms of glucose, peptone, and yeast extract), a similar mechanism could have occurred. Organic matter consumption produces CO_2_ and NH_4_^+^, which is totally or partially utilized for the bioformation of carbonates. Since no precipitation was observed in the control experiments of noninoculated media, the importance of microbial activity in bioprecipitation processes was demonstrated. Accordingly, this data suggests that nutrient composition or nutrient concentration in brine used was not enough for biominerals precipitation. Nevertheless, crystal formation was successful in the organic matter-amended brine. Finally, when the carbon source was peptone, no carbonate precipitation was observed.

Castanier et al. (1999) [32], as well as McConnaughey and Whelan (1997) [33], proposed that ion transport (especially Ca^2+^) across cellular membrane is linked to bacteria-mediated crystal precipitation. In* Bacillus* and* Virgibacillus*, the calcium concentration progressively decreased over time, reaching percentages of 25% after 20 days of culture. However, the percentages of magnesium reduction were always lower than the calcium percentages with values ranging to 80% after 20 days of culture. Greater power for ionic selectivity produces more adsorption of Ca^2+^ than of Mg^2+^ in the bacteria cellular envelope [34, 35]. As pointed out by Rosen (1987) [36], the bacterial Ca^2+^ pump is displaced close to the outside of the cell, whereas the Mg^2+^ pump is located towards the inside. It has been defended that extracellular Ca^2+^ concentrations are around 103-fold higher than intracellular Ca^2+^ concentrations [37, 38]. These findings explain the greater tendency of these bacterial strains to precipitate calcium carbonate or calcium sulphate and calcium carbonate instead of magnesium minerals, even when the concentration of magnesium ions in the brine was much higher than the concentration of calcium ions.

**Figure 3:**
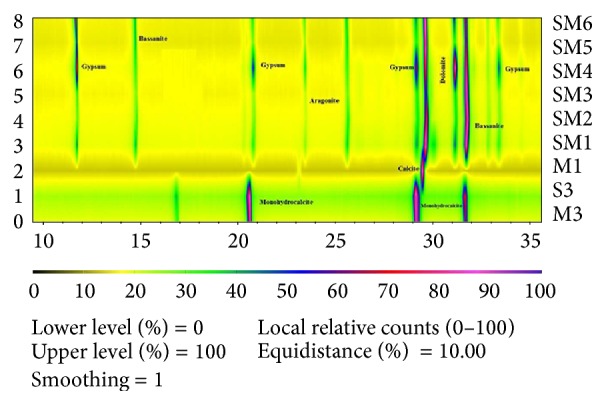
X-ray map of the precipitate formed in the solid medium (SM1, SM2, SM3, SM4, SM5, SM6, and M1) and SM2 liquid medium inoculated with the strain S3 and strain M3. Calcite CaCO_3_; dolomite Ca Mg (CO_3_)_2_; aragonite CaCO_3;_ gypsum CaSO_4_·H_2_O; bassanite 2CaSO_4_·H_2_O; and monohydrocalcite CaCO_3_·H_2_O.

Figure 3 (X-ray map of the precipitate formed) and Table 5 (semiquantitative analysis of precipitates) show the results of crystal formation of the six selected strains grown in M1, SM1, SM2, SM3, SM4, SM5, and SM6 solid media. In this context, X-ray diagrams showed that precipitated crystals were 100% calcite in M1 medium and calcium sulphate (gypsum and bassanite) and calcium carbonate (aragonite, dolomite, and calcite) in the rest of the culture media. In this sense, when yeast extract was added to the culture media, carbonate crystals were formed. Finally, in culture media containing protease peptone and glucose, most of the crystalline precipitates were composed of gypsum and bassanite. However, carbonate crystals such as monohydroxycalcite (CaCO_3_·H_2_O) were formed by* Virgibacillus pantothenticus* S3 and Bacillus marisflavi M3 in liquid medium after 30 d of growth.

Several researches have demonstrated that bacteria can become the nucleus of mineral precipitation due to cellular surface membrane, cell wall, or EPS layers cation adsorption [16, 39–41]. Hammes and Verstraete (2002) [42] observed that thin, watery layers around bacterial cells create microenvironments subjected to different pH and dissolved organic carbon concentrations, in which calcium cations can prevail. Our results suggested that precipitation of different minerals is induced by the different cellular envelopes of the different bacteria found in this study. These results are similar to those of Rivadeneyra et al. (2004) [18], who showed that different bacterial strains can create microenvironments with different concentrations of calcium and magnesium cations and therefore result in different characteristics of precipitated minerals. They also agreed with Schultze-Lam et al. (1996) [43], who also claimed that the electronegative nature of the cell membrane can set up a unique precipitation environment on a microscale.

The PHREEQC results (Table 6) are presented in terms of the saturation index (SI) for each mineral. SI is defined by SI = log (IAP/Ksp), where IAP is the ion activity product of the dissolved constituents and Ksp is the solubility product for the mineral. Thus, SI < 0 indicates undersaturation with respect to the mineral, while SI > 0 indicates supersaturation. The geochemical study indicated that the ionic conditions of the media were suitable for the generation of vaterite, hydroxyapatite, aragonite, calcite, dolomite, huntite, magnesite, and struvite. When the selected strains were grown in M1, only calcite was precipitated. However, in SM1, SM2, SM3, and SM4 solid media, carbonates such as dolomite, aragonite, and calcite and calcium sulphates such as bassanite and gypsum were formed. In addition, in SM5 and SM6 solid media, only bassanite and gypsum were precipitated. Also, in SM2 liquid medium inoculated with strains M3 and S3, monohydrocalcite was the only mineral precipitated. Finally, vaterite, hydroxyapatite, huntite, and struvite were never detected in our experiments, despite being, from a geochemical viewpoint, the most often formed minerals.

In the results of the geochemical analysis software PHREEQC, vaterite, hydroxyapatite, aragonite, calcite, dolomite, huntite, magnesite, and struvite could possibly be inorganically precipitated in the solutions since their SI was greater than 0 (Table 6). However, when the selected strains were cultivated in culture media containing real brine from a desalination plant, lower amounts of carbonates such as dolomite, aragonite, and calcite were produced compared to the amounts of sulphates such as bassanite and gypsum formed. These results show that, in media containing real brine with a high concentration of sulphates (Table 2), the bacteria promote the biomineralisation of sulphates compared to carbonates. Probably, this fact is a consequence of the complex ion composition of the brine and also of the different ion interactions produced in this habitat. Moreover, our data suggest that high concentrations of sulphate may compete with the carbonate for the calcium ions present in the brine. This fact can be observed in Table 5, where the importance of concentration and type of organic matter on the precipitation of carbonate and sulphates minerals is evident. Also, water solubility of CO_2_ depends on temperature, pressure, pH, and brine salinity [2]. These environmental parameters need to be understood given that they determine bioprecipitation process and CO_2_ sequestration in brines. Consequently, modification of these environmental parameters could affect the precipitation of biominerals.

**Figure 4:**
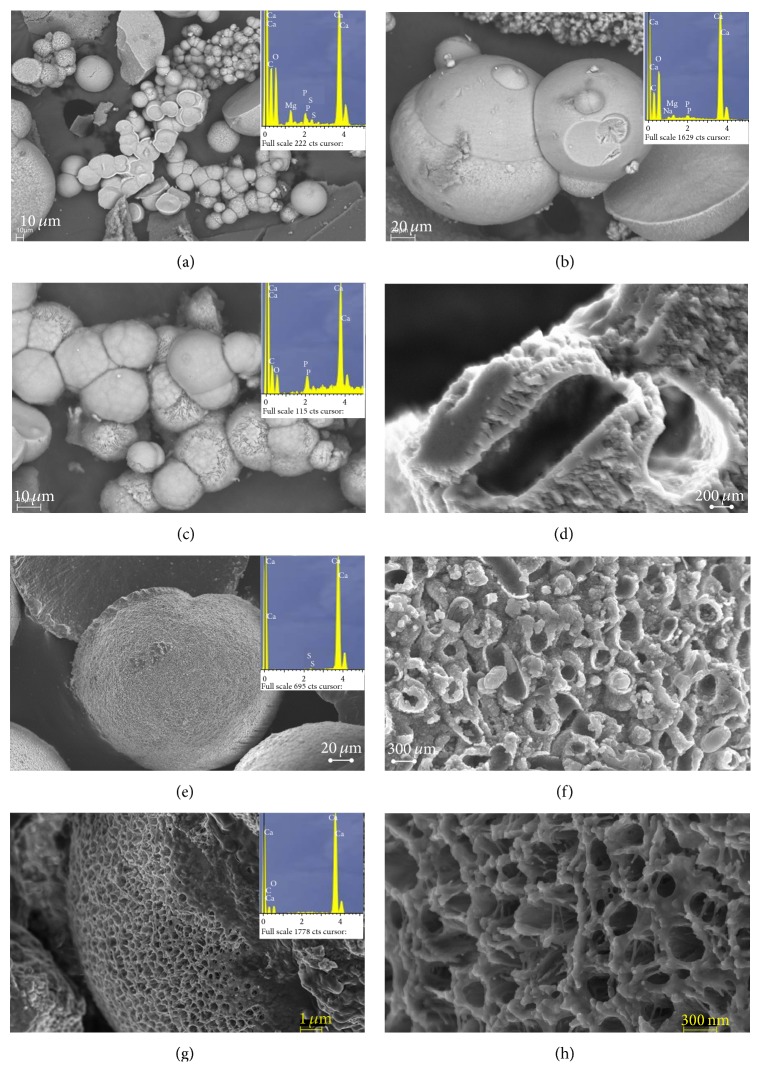
Scanning electron micrographs (SEM and FESEM) of calcium carbonate crystals precipitated. (a), (b), (c), and (e) show bioliths produced in solid media in different stages of formation with morphologies of spheres and hemispheres and EDX spectrum; some bioliths show bacteria fingerprints on the surface. (d) and (g) are detail of the surface of some bioliths. (d) shows the surface of one calcified cell. (f) shows detail of (e), where the abundance of calcite nanoparticles delimiting the bacterial cell contours is evident. (g) and (h) show bioliths of monohydrocalcite precipitates in liquid medium and EDX spectrum. (h) shows detail of the biolith surface.

**Figure 5:**
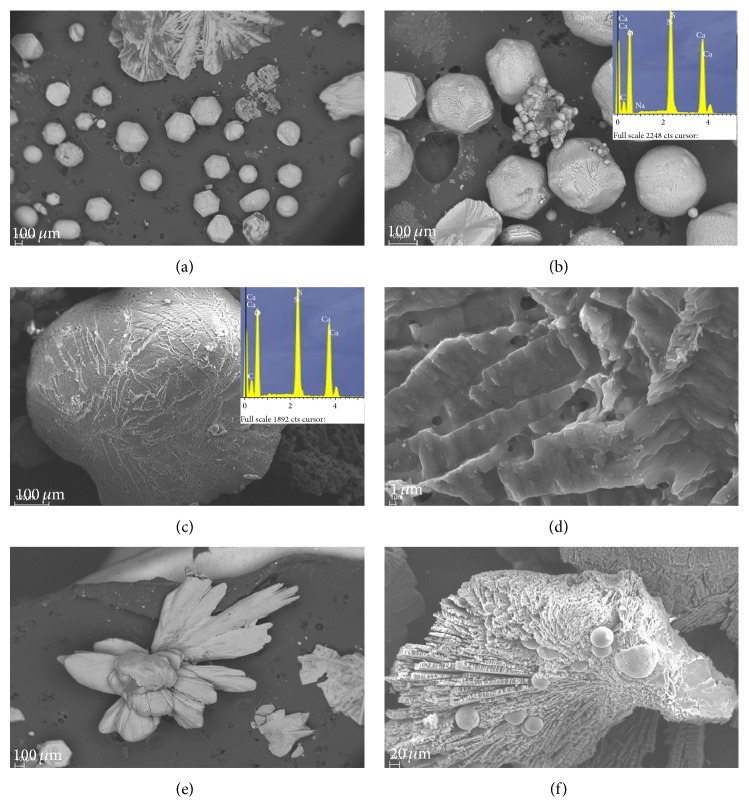
Scanning electron micrographs (SEM and FESEM) of calcium sulphate crystals precipitated in solid media. (a), (b), and (c) show polyhedral and pseudospheres shapes and EDX spectrum. (a) and (b) also show some spherulites of carbonate. (d) is detail of (c), where the surface of a biolith of sulphate mineral without mineralised cells can be observed. (e) and (f) show different morphologies of sulphate bioliths. (e) shows small spheres of calcium carbonate.

The XRD analysis confirmed the bioprecipitation in all of the culture media, irrespective of the strain tested. A variety of shapes was observed in both types of mineral study. Particularly in the case of carbonate minerals, the most important morphologies were spheres and hemispheres forms, which appeared either in isolation or in groups. Most of the carbonate crystals formed in all culture media had surfaces with small holes, and high porosity (Figure 4). Shape and size of many holes resembled those of bacteria, and mineralised cells were frequently evident. However, calcium sulphate crystals had pseudospheres, polyhedral shapes, and crystal twinning (Figure 5). Finally, mineralised cells were never detected on the surfaces of the minerals.

The SEM of the carbonate crystals shows bioliths with spheroidal morphologies in the cultures (Figure 4). In many cases, mineralised bacteria are evident on spherulite surfaces, and bacterial mould entirely covers the surfaces of certain bioliths (Figures 4(a), 4(d), 4(f) and 4(h)). This confirms that the crystals were formed by an accumulation of calcified organisms. More detailed images of the spherulite surfaces (Figure 4(d)) showed that carbonate precipitates were nucleated on bacterial nanoglobules. These nanoglobules occurred in the external bacterial envelope. Carbonate nanoglobule formation is thought to be the first stage of microbially-mediated precipitation processes [44]. The observation of nanoglobules and calcified cells are clear evidence that the bacteria accumulate and precipitate carbonates on the cell surface during bacterial growth.

It has been shown that carbonate precipitation mediated by moderately halophilic bacteria* Chromohalobacter marismortui* started with the early appearance of amorphous calcium phosphate, nanoparticles formed by the union of Ca^2+^ ions, and the phosphate groups of the outer membrane and cytoplasmic membrane components [16]. The transformation of phosphates into amorphous calcium carbonate during maturation of spherulites led to formation of aragonite and increase in crystallinity. In this sense, EDX analysis performed on different bioliths suggests this formation mechanism; the presence or absence of phosphate during the formation and maturation process of bioliths is evident (Figure 5).

The bioliths, formed by the union of calcified cells, increase in size as new calcified cells are progressively added. In the same way, our study suggested that mineralisation of the external envelopes of the bacteria (Figure 5) produces cell death since it inhibits an exchange of substances with the environment and causes the cells to break down; the release of the intracytoplasmatic contents may provide nutrients for other cells in the population and also contribute to recrystallisation processes in bioliths. Moreover, according to the hypothesis of programmed cell death, a percentage of the population will be genetically programmed to commit suicide. It has thus been observed that, in certain situations of extreme stress, a significant part of the population may commit mass suicide. This is for the greater good, since the death of these individuals guarantees the survival of other members, who will thus be able to resist until conditions improve [45]. In this context, in environments with a high calcium concentration (especially of Ca^2+^), such as brines, carbonate precipitation may very well be a survival mechanism, which allows certain individuals of the population to survive, thanks to the sacrifice of the other members, as previously reported by Silva-Castro et al. (2013) [46]. If this hypothesis is confirmed, it would add an interesting dimension to the study of bacterially mediated carbonate precipitation and concomitantly to CO_2_ capture. This would mean that, in addition to its ecological importance in carbon and calcium cycles, it would also be a key element that enables the survival of bacteria populations.

The most important finding of this research study is probably the fact that the heterotrophic bacterial community from saline environments (seawater and real brine) showed the potential ability to precipitate biominerals such as aragonite, calcite, dolomite, gypsum, and bassanite in culture media amended with a high concentration of real brine from desalination plants. However, precipitation only occurred when the microbiota were grown in environments with concentrations of organic matter and never when the microbiota were grown in environments derived from real brines without the addition of organic matter. This result suggests that, in the brines used in our experiments, the precipitation of biominerals, such as calcium carbonate, through autochthonous microbiota action cannot take place* in situ*, as a consequence of the low organic matter concentration present in this waste. However, if the brines are added with a sufficient concentration of organic matter, then biomineralisation could be produced. Consequently, if this circumstance is produced, the biosequestration of CO_2_ can take place. The search for alternative sources of organic matter that can be used in these processes is a challenge for future research.

## Figures and Tables

**Table 1:** Chemical composition of the soil media used in the study.

Medium	Yeast extract (g/L)	Protease-peptone (g/L)	Glucose (g/L)
SM1	10	5	1
SM2	7.5	3.75	0.75
SM3	7.5	—	2
SM4	7.5	—	3
SM5	—	5	2
SM6	—	5	3

All of the culture media were prepared with real brine as mineral source.

**Table 2:** Ionic composition (mg/L) of different solid and liquid media used in this study. The liquid medium used in the experiment was SM2 medium which was inoculated with strain M3 (*Bacillus marisflavi*) or strain S3 (*Virgibacillus pantothenticus*).

Compounds	Solid medium							Liquid medium	
	M1	SM1	SM2	SM3	SM4	SM5	SM6	M3	S3
Ca^+2^	1040	770	770	770	770	770	770	556	535
Mg^+2^	890	2340	2340	2340	2340	2340	2340	213	103
Na^+^	756	11800	11800	11800	11800	11800	11800	11800	11800
K^+^	260	450	450	450	450	450	450	450	450
SO_4_^−2^	1910	3166	3166	3166	3166	3166	3166	3166	3166
Cl^−^	1504	21100	21100	21100	21100	21100	21100	21100	21100
TC	6484	6484	4863	2386	2003	2920	3333	1828	1678
NH_4_^+^	1728	1728	1296	651	434	642	642	487	447
P	150	150	112	90	60	0	0	42	39
pH	8.4	8.2	8.1	8.0	8.2	8.1	8.0	7.9	7.8

TC: total carbon.

**Table 3:** Taxonomic identification of selected strains through partial 16S rRNA gene sequence.

Strain		Accession number	Gene sequence of 16s rRNA	% identity	Sequence length (bp)
Brine	S1	KP721634	NR_025240 *Bacillus marisflavi *	91	593
	S2	KP721635	NR_118437 *Bacillus marisflavi *	95	1466
	S3	KP721636	NR_114091 *Virgibacillus pantothenticus*	89	1382

Seawater	M1	KP721631	NR_074977.1 *Bacillus pumilus *	99	1505
	M2	KP721632	NR_074977.1 *Bacillus pumilus *	99	1501
	M3	KP721633	NR_118437.1 *Bacillus marisflavi *	97	1396

**Table 4:** Carbonate mineral precipitation in solid media and carbonic anhydrase activity.

Strain	Number of days strains take to precipitate						Carbonic anhydrase
	Culture media tested						
	SM1	SM2	SM3	SM4	SM5	SM6	
*Bacillus marisflavi *S1	4	6	NF	8	14	14	NR
*Bacillus marisflavi *S2	4	6	NF	10	NF	NF	NR
*Virgibacillus pantothenticus* S3	2	2	8	8	14	14	NR
*Bacillus pumilus *M1	6	8	NF	8	NF	NF	PR
*Bacillus pumilus *M2	6	10	8	10	16	14	PR
*Bacillus marisflavi *M3	2	2	8	8	14	14	PR

PR: positive reaction; NR: negative reaction (no yellow colouration). NF: non-crystal-formation.

**Table 5:** Semiquantitative analysis (%) of precipitates produced in solid media cultures containing brine as calcium source by the six selected strains.

Medium	Aragonite	Bassanite	Calcite	Dolomite	Gypsum
SM1	02.8	74.3	01.4	08.5	13.2
SM2	02.3	84.5	00.0	06.3	06.8
SM3	02.8	80.2	00.0	07.7	09.3
SM4	04.1	48.8	01.5	19.3	26.2
SM5	00.5	79.4	00.0	00.0	20.1
SM5	00.0	70.4	00.0	00.0	30.6

**Table 6:** Saturation index values (SI) for mineral formation in culture solid medium (M1, SM1, SM2, SM3, SM4, SM%, and SM6) and liquid medium SM2 inoculated with strains S3 and M3. All of the culture media contained added artificial brine.

Mineral	Solid medium							Liquid medium	
	M1	SM1	SM2	SM3	SM4	SM5	SM6	S3	M3
Anhydrite	−0.70	−0.72	−0.69	−0.65	−0.64	−0.65	−0.65	−0.63	−0.63
Aragonite	2.43	2.25	2.15	1.87	1.80	1.95	2.01	1.63	1.68
Calcite	2.57	2.40	2.29	2.01	1.94	2.10	2.15	1.78	1.83
Dolomite	5.47	5.69	5.47	4.91	4.77	5.08	5.19	3.23	3.63
Dolomite (d)	4.92	5.14	4.92	4.36	4.22	4.53	4.64	2.68	3.08
Gypsum	−0.49	−0.52	−0.49	−0.45	−0.44	−0.45	−0.45	−0.43	−0.43
Huntite	6.92	7.92	7.49	6.36	6.08	6.72	6.93	1.79	2.88
Hydroxyapatite	13.41	12.18	11.85	11.65	11.14	—	—	11.4	11.41
Magnesite	2.32	2.71	2.60	2.32	2.25	2.41	2.46	0.87	1.22
Monohydrocalcite	−1.83	−2.00	−2.11	−2.39	−2.46	−2.3	−2.25	−2.62	−2.57
Nesquehonite	−0.12	0.26	0.16	−0.12	−0.19	−0.03	0.02	−1.57	−1.22
Struvite	2.39	2.64	2.38	1.99	1.63	—	—	0.62	0.95
Vaterite	18.7	18.53	18.42	18.14	18.07	18.22	18.28	17.91	17.95
